# A Comprehensive Review on Beneficial Effects of Catechins on Secondary Mitochondrial Diseases

**DOI:** 10.3390/ijms231911569

**Published:** 2022-09-30

**Authors:** Baoyi Chen, Wenting Zhang, Chuyuan Lin, Lingyun Zhang

**Affiliations:** College of Horticulture, South China Agricultural University, Guangzhou 510640, China

**Keywords:** catechins, secondary mitochondrial diseases, biogenesis, calcium homeostasis, indirect beneficial

## Abstract

Mitochondria are the main sites for oxidative phosphorylation and synthesis of adenosine triphosphate in cells, and are known as cellular power factories. The phrase “secondary mitochondrial diseases” essentially refers to any abnormal mitochondrial function other than primary mitochondrial diseases, i.e., the process caused by the genes encoding the electron transport chain (ETC) proteins directly or impacting the production of the machinery needed for ETC. Mitochondrial diseases can cause adenosine triphosphate (ATP) synthesis disorder, an increase in oxygen free radicals, and intracellular redox imbalance. It can also induce apoptosis and, eventually, multi-system damage, which leads to neurodegenerative disease. The catechin compounds rich in tea have attracted much attention due to their effective antioxidant activity. Catechins, especially acetylated catechins such as epicatechin gallate (ECG) and epigallocatechin gallate (EGCG), are able to protect mitochondria from reactive oxygen species. This review focuses on the role of catechins in regulating cell homeostasis, in which catechins act as a free radical scavenger and metal ion chelator, their protective mechanism on mitochondria, and the protective effect of catechins on mitochondrial deoxyribonucleic acid (DNA). This review highlights catechins and their effects on mitochondrial functional metabolic networks: regulating mitochondrial function and biogenesis, improving insulin resistance, regulating intracellular calcium homeostasis, and regulating epigenetic processes. Finally, the indirect beneficial effects of catechins on mitochondrial diseases are also illustrated by the warburg and the apoptosis effect. Some possible mechanisms are shown graphically. In addition, the bioavailability of catechins and peracetylated-catechins, free radical scavenging activity, mitochondrial activation ability of the high-molecular-weight polyphenol, and the mitochondrial activation factor were also discussed.

## 1. Introduction

Mitochondria are important organelles for eukaryotic energy metabolism and are the main sites for oxidative phosphorylation of cells to produce ATP. The mitochondria participate in the physiological functions of cell energy metabolism, maintaining ion concentration gradient, and transmitting apoptosis signals [1]. Mitochondrial abnormalities can lead to cell and even organ damage, and a growing number of diseases are associated with mitochondrial defects [2]. Mitochondrial defects are a clinically heterogeneous group of disorders caused by a series of mutations in genes encoded by the mitochondrial or nuclear genome [3]. The mitochondrial diseases comprise various disorders that can exhibit a series of combination of clinical features [4].

It is estimated that these diseases affect about one in every 5000 people worldwide, and are usually the most common inherited metabolic diseases [5]. Mitochondrial diseases are now widely regarded as a critical cause of genetic disease. They exhibit prominent biochemical, phenotypic, and molecular genetic heterogeneity, and typically involve multiple organ systems [6]. Mitochondria are present in all nucleated cells. Mitochondria are unique organelles among all intracellular organelles because they contain their own DNA. Through the migration, isolation, and evolution of humans, mitochondrial DNA (mtDNA) formed a wide range of mitochondrial genome polymorphisms [7]. Mitochondria are complex organelles encoded both by nuclear and mtDNA [8]. A large number of causative genetic defects, which are localized to the nuclear genome or the mitochondrial genome, have been identified [9]. Therefore, genetic defects in mitochondrial or nuclear DNA may impair ATP production and may potentially lead to human pathology at any age, with any symptoms, and by any genetic mode [10]. Tissues which are highly dependent on oxidative metabolism, such as the heart, brain, skeletal muscle, renal tubules, retina, and endocrine glands, have a lower threshold for onset. Therefore, these tissues are particularly vulnerable to pathogenic mutations in mitochondrial DNA [11]. Primary mitochondrial diseases occur not only due to defective genes encoding electron transport chain proteins, but also due to germline mutations in other nuclear DNA genes that affect oxphos function by impacting production of the complex machinery needed for the electron transport chain to perform optimally [12]. In contrast, secondary mitochondrial diseases can accompany many pathologic processes not involving oxphos, including inherited diseases with germline mutations in non-oxphos genes. SMD can also be acquired secondary to adverse environmental effects which can cause oxidative stress [13,14,15]. Thus, secondary mitochondrial diseases can be inherited or acquired, which is an important distinction from primary mitochondrial diseases, which can only be inherited. In this review, we will mainly discuss the latest findings regarding secondary mitochondrial diseases rather than primary mitochondrial diseases.

Polyphenols are a large group of secondary metabolites extensively distributed in plant products, and have been implicated in improving human health by reducing chronic disease [16]. Catechins are flavanol compounds of the polyphenols family, which is an important constituent of tea flavor and soup color [17]. According to stereochemistry, catechins can be divided into two groups: (+) forms (2S, 3R) catechins and (−) epi forms (2R, 3R) catechins. Most of the catechins in fresh tea leaves and green tea are in the (−) epitope form (2R, 3R) [18,19]. Catechin compounds are the main functional components in tea, accounting for 12% to 35% of the dry weight of tea. The catechin compounds in tea tree mainly include epigallocatechin gallate (EGCG), epicatechin gallate (ECG), epigallocatechin (EGC), epicatechin (EC), gallocatechin gallate (GCG), catechin gallate (CG), gallocatechin (GC), and catechin (C) [20].

The catechin compounds which are rich in tea have attracted much attention due to their effective antioxidant activity. Various physiological activities of catechins have been fully confirmed in cell and animal models, but the lack of clinical research greatly limits the clinical application of catechins. With the development of modern medicine and biotechnology research, the mechanism of action of catechins on various chronic diseases has become increasingly obvious, and its pharmacological development and utilization have become increasingly clear. At present, the roles and mechanisms of catechins have not been comprehensively or systematically introduced. This paper reviews the latest research progress of catechins in mitochondrial diseases in recent years, and provides a theoretical basis for future research on catechins.

## 2. Attenuation of Secondary Mitochondrial Diseases by Catechins

SMD does not involve inborn defects of genes controlling oxphos, and usually presents after conception. The impact of another pathologic process on mitochondria, as seen in many inherited and acquired disorders (non-PMD disorders), can secondarily attenuate mitochondrial ability to generate ATP and alter mitochondrial dynamics, which can impact the non-ATP producing capabilities as well. For instance, mitochondrial fission and fusion, as examples of mitochondrial dynamics, are often implicated in multifactorial disorders such as diabetes [21], heart disease [22], cancer [23], kidney diseases [24], and neurodegenerative disorders [25]. Therefore, these complex disorders are frequently accompanied by SMD [26]. In recent years, due to its properties of alteration of mitochondrial dynamics and prevention of the progression of chronic diseases, more and more studies have focused on beneficial effects of catechins against representative secondary mitochondrial diseases (such as metabolic disorders, degenerative diseases, etc.).

### 2.1. The Neurodegenerative Diseases

Neurodegenerative diseases are progressive degeneration states in which cells and neurons of the brain and spinal cord are lost. They are a group of diseases that result from the loss of neurons or their myelin sheaths, which results in a decline or loss of function over time—for example, Alzheimer’s disease (AD), amyotrophic lateral sclerosis (ALS), Parkinson’s disease (PD), Huntington’s disease (HD), etc. The reduction in or loss of mitochondrial function and the oxidative damage caused by free radicals play a major role in promoting and aggravating the occurrence and development of chronic and programmed cell death and diseases [27]. Namely, persistent mitochondrial damage causes chronic inflammation, and in the central nervous system, neuronal cell death caused by chronic inflammation may be a major cause of neurodegenerative diseases [25].

#### 2.1.1. Alzheimer’s Disease

AD is one of the most typical neurodegenerative diseases [28]. The principal pathological mechanism of AD is accumulation of amyloid β (Aβ) and neurofibrillary tangles [29]. Mitophagy is a mechanism that selectively removes damaged mitochondria through autophagy during cell evolution, and timely removal of damaged mitochondria plays a significant role in maintaining the normal physiologic functions of cells. In neurons of AD patients, when amyloid-β and neurofibrillary tangles accumulate, mitochondria are damaged, and severely damaged mitochondria can be removed by selective mitophagy. When mitophagy is blocked, significant dysfunctions such as mitochondrial transport and abnormal dynamics appear in neurons, leading to aggravated pathological changes in Alzheimer’s disease. Thus, in models of Alzheimer’s disease, enhancing mitophagy could inhibit aggregation of amyloid-β and neurofibrillary tangles, and reverse cognitive deficits [30].

Research shows that flavonoids extracted from plants have the function of improving AD phenotypes; however, the mechanism for protecting from AD is still unclear [31]. A recent study suggested that catechins suppressed amyloid-β and neurofibrillary tangles, showing potential prevention and treatment of AD [32]. Fernando has confirmed that catechins can influence the formation of amyloid-beta and increase patients’ cognitive function [33]. Studies showed that EGCG might convert amyloid-beta into smaller, amorphous protein aggregates [34]. Jan Bieschke’s research appears to confirm that green tea EGCG can restore damaged mitochondrial membrane potential, ATP levels, or reactive oxygen species production, which decrease Alzheimer’s Aβ-induced mitochondrial dysfunction [31]. In addition, EGCG can also inhibit Aβ-induced oxidative stress and neuroinflammation; that is, it can reduce amyloid-β-induced neuroinflammatory response by reducing amyloid-β-induced oxidative stress [35].

#### 2.1.2. Parkinson’s Disease (PD)

PD is also a multifactorial disorder whose etiology is induced by genetic and environmental factors. However, some evidence shows that iron accumulation, oxidative stress, and mitochondrial dysfunction are the central pathogenesis [36].

PD is mostly sporadic, but several inherited cases are caused by mutations in single genes [37]. Rare PD is triggered by mutations in nuclear genes, and there is growing evidence that susceptibility genes increase the risk of disease [38]. With the advancement of research, multiple pathogeneses of PD have been proposed, in which mitochondrial dysfunction plays a critical role in the familial forms and sporadic of the diseases [39]. In some patients with sporadic critical, a key feature is respiratory chain damage, and evidence suggests that proteins encoded by Parkinson’s disease-associated genes are closely related to mitochondrial dysfunction [40].

Research suggests that PD is related to systemic deficits in mitochondrial complex I activity [38]. Complex I deficiency may lead to oxidative stress and increase neuronal susceptibility to excitotoxic death. EGCG can reduce neurotoxin-induced oxidative stress damage in a mice PD model [41]. In some neurodegenerative diseases, the main chemical pathology observed is the accumulation of iron ions at sites where there is neuronal inactivation [42]. Some studies have shown that iron excess caused by excessive iron absorption or utilization defects can easily lead to abnormal iron deposition in tissues, which will eventually lead to oxidative damage [43]. Iron ions exist in Lewy bodies and other amyloid structures in ferrous (Fe^2+^) and ferric (Fe^3+^) states, and Lewy bodies are also one of the pathological features of PD [44].

Mitochondria are the main source of pro-oxidants and the main intracellular receptors for iron ions [36]. Kinetic experiments showed that extracellular iron ions were easily transported into mitochondria. Several epidemiological studies show benefits of tea drinking for PD [45,46,47]. It was reported that catechins had potent free radical scavenging, chelating iron, and anti-inflammatory activities, and could protect from neuronal death in animal and cellular models of neurological disease [48]. The intracellular metal chelating ability of catechins may prevent iron-induced oxidative stress and amyloid β aggregation of peptides [49].

In the structure of EGCG, the important chelating groups are the 3,4-dihydroxyl and gallic acid groups in the B ring, which can neutralize ferric ions to form redox state inactive iron, thereby protecting neuronal cells from immune damage [50,51]. Since the gallate group in catechins is a metal binding site, catechins can prevent metal ion homeostasis from causing oxidative stress, which is associated with preventing the occurrence of chronic diseases, e.g., Parkinson’s disease [52].

#### 2.1.3. Huntington’s Disease (HD)

HD is a progressive, neurodegenerative disease characterized by abnormal involuntary movements, progressive dementia, and psychiatric disturbances [53]. The disease is triggered by the expansion of CAG triplet repeats in the polyglutamine region of the huntingtin protein, which leads to intracellular huntingtin protein aggregation. Therefore, small molecule compounds that prevent or reverse these protein misfolding events may have therapeutic effects [54]. Studies have shown that the levels of iron and copper ions are increased in the definite brain areas of Huntington’s disease patients. Epigallocatechin gallate, as an iron and copper chelator, may modulate early disturbances in huntingtin folding [55]. Studies have shown that administration of EGCG reduces the toxicity of the huntingtin protein in yeast and fruit fly models [56]. EGCG can directly bind to unfolded polypeptides and redirect their aggregation to the unfolded pathway, resulting in the formation of harmless, unstructured, and highly stable oligomers [57].

As with Alzheimer’s disease and Parkinson’s disease, substantial evidence suggests that mitochondrial failure is closely related to the pathogenesis of Huntington’s disease, and degenerating striatal neurons in HD are significantly susceptible to complex II inhibition [58]. EGCG (20 mg/kg) significantly attenuated behavioral changes, mitochondrial complex enzyme dysfunction, oxidative damage, and striatal damage in 3-nitropropionic acid treated animals [59]. Accumulating evidence supports the notion that EGCG polyphenolic flavonoids have a protective effect on neuro-degeneration. EGCG can not only influence the pathogenic mechanisms of underlying neurodegenerative disorders, but can also have a prominent ability to slow the progression of the disease [55].

Due to their ability to donate electrons and hydrogen atoms, catechin compounds have shown various potential neuroprotective properties [60]. Epicatechin and EGCG are useful as neuroprotective agents, mainly due to their advantage of crossing the blood-brain barrier [61]. Cell culture studies have confirmed that catechins protect against neuronal cell death caused by a variety of neurotoxins, such as 1-methyl-4-phenylpyridine, 6-hydroxydopamine), and amyloid-β peptide [62,63,64,65]. In summary, the neuroprotective effects of EGCG stem from its ability to scavenge free radicals, inhibit protein aggregation, inhibit/enhance amyloid/non-amyloid hydrolysis processes, chelate iron, and synergistically modulate various other cellular pathways.

### 2.2. Metabolic Disorders

#### 2.2.1. Diabetes Mellitus

Diabetes mellitus is a threatening metabolic syndrome that occurs when the pancreas cannot produce enough insulin, or when the body is no longer able to make much of the insulin it produces [66]. According to the global diabetes associations, about 642 million people will be living with diabetes in worldwide by 2030. A continued global increase in the prevalence of type 2 diabetes (T2D) has been reported, which accounted for around 90% of all diabetes [67].

Recent research using animal models of diabetes suggest that mitochondrial dysfunction plays an significant role in diabetes-induced neurodegeneration [68]. The results showed that in some cases, mitochondrial dysfunction was critical for the development of insulin resistance and diabetes, while in other cases, mitochondrial dysfunction accelerated and exacerbated the pathophysiology of diabetes [69]. Much data suggests that correct mitochondrial function is crucial for maintaining glucose-induced insulin secretion [70].

Studies have shown that proinflammatory cytokine-mediated pancreatic β-cell dysfunction is a significant pathological feature in type 1 diabetes. More and more research demonstrates that the mitochondria play a critical role in regulating the insulin releasing capacity of β-cells. Beta-cell dysfunction effects can be restrained by EGCG treatment through the mitochondrial pathway [71]. In addition, a previous study found that drinking purified water containing 0.05% EGCG might effectively delay the onset of type 1 diabetes mellitus (T1D) in non-obese-diabetic mice [72]. Oolong tea consumption can effectively lower the plasma glucose levels in T2D patients [73].

In some animal experiments, long-term intake of tea catechins may help to suppress obesity by regulating metabolism of lipids, thereby improving lipid and glucose metabolism disorders caused by T2D [74]. Among all green tea catechins, EGCG has a significant antidiabetic effect in the model of T2D. In addition, supplemental intake of EGCG in patients may potentially improve glucose tolerance in type 2 diabetes [75]. Studies have also shown that catechins can effectively control hyperglycemia and prevent diabetes complications by improving insulin sensitivity as well as reducing risk factors for T2D, such as dyslipidemia, obesity, and oxidative stress [76].

Epidemiological studies have confirmed that drinking tea can greatly decrease the risk of diabetes and its complications. For some time, it has been found that drinking of green tea has anti-diabetic effects, and the mechanism may be that tea catechins can decrease glucose concentration and increase glucose tolerance in the body [77]. In cell culture and animal experiments, catechins have the following potential mechanisms of action for the treatment of diabetes: inhibiting glucose absorption by inhibiting α-glucosidase and interaction with glucose transporters, reducing hepatic glucose production, enhancing pancreatic function, and protection of pancreatic β cells by inhibiting nuclear factor κB (NF-κB) activation [78].

#### 2.2.2. Metabolic Complications

As a typical chronic metabolic disease, diabetic patients may have many serious complications. Diabetic cardiomyopathy and nephropathy are typical complications of diabetes. In recent years, catechins or green tea extracts have been used to protect against diabetic complications by animal models. The pathogenesis of diabetes complications and the possible inhibitory mechanism of catechins are briefly described as follows.

##### Diabetic Cardiomyopathy (DCM)

Mitochondria are the energy factories of cells and maintain the stability and performance of cardiomyocytes [79]. According to statistics, the incidence of cardiomyopathy in patients with mitochondrial disease is about 20–25%, and in some series it is as high as 40% [79]. DCM is defined as the changes in the structure and function of the myocardium that occur in a diabetic milieu independent of the coexistence of other confounding factors [40]. The pathogenesis of DCM is complex, and is characterized by hyperglycemia, dysregulated lipid metabolism, insulin resistance, mitochondrial dysfunction, and disturbances in adipokine secretion and signaling. These abnormalities lead to impaired calcium homeostasis, ultimately resulting in lusitropic and inotropic defects [80]. Accumulating evidence indicates that hyperglycemia is one of the main pathological mechanisms in DCM [81,82,83]. However, the long-standing hyperglyceia induces some metabolic and molecular changes in the myocardial cells [84]. These metabolic changes result in the generation of reactive oxygen species (ROS) from mitochondria, as well as a reduction in capacity of the key antioxidant enzyme glutathione reductase and formation of advanced-glycation end-products (AGEs) [85]. Consequently, the oxidative balance is disturbed by the increased oxidative stress that, in turn, leads to DNA damage and contributes to accelerated cardiomyocyte death [86].

Extensive evidence reveals that mitochondrial dysfunction plays a critical role in the development of DCM [87]. Mechanisms underlying the mitochondrial dysfunction and impairment in mitochondrial morphology include altered energy metabolism-induced mitochondrial uncoupling, oxidative stress, impaired mitochondrial Ca^2+^ handling, transcriptional and translational alterations of oxidative phosphorylation, and altered mitochondrial dynamics and biogenesis [88]. In addition, hyperglycemia causes dynamic changes of mitochondrial morphology of the neonatal cardiomyocyte cell line and results in overproduction of ROS [89]. Catechins are the major powerful and pharmacologically active compounds in tea. The potential therapeutic effect of EGCG on DCM mainly contributes to its hypoglycemic, hypolipidemic, maintenance calcium homeostasis, and ROS scavenging properties. There is a considerable evidence showing the antidiabetic effect of EGCG in vitro and in vivo [90,91]. EGCG inhibits glucose production in isolated hepatocytes through suppression of hepatic gluconeogenesis and activation of 5’-AMP-activated protein kinase (AMPK) [92]. EGCG is also able to decrease glucose production from rat H4IIE hepatoma cells by increasing the insulin receptors and regulating the genes that encode gluconeogenic enzymes [93].

In addition, EGCG was found to reduce the plasma levels of glucose by inhibiting α-glucosidase activity [94]. EGCG was found to inhibit sodium-dependent glucose transporter-1 in the intestine, thereby preventing glucose uptake [95]. Furthermore, EGCG exerted an insulin mimetic activity by decreasing the level of mRNAs for gluconeogenic enzymes, including PEPCK and glucose-6-phosphatase in the mouse liver [96].

Chronic dyslipidemia has emerged as a major factor in the pathogenesis of cardiac complications and atherosclerosis [97]. A study demonstrated treatment with EGCG attenuated serum glucose, TC, TG, HDL-C, and LDL-C levels in STZ-diabetic rats [98]. A previous study showed that EGCG treatment markedly reduced serum levels of TC, TG, VLDL-C, LDL-C, and HDL-C in Wistar rats. In another study, treatment of hypercholesterolemic rats with EGCG reduced levels of TC, TG, LDL-C, and VLDL-C levels in serum and decreased the cardiac risk ratio [99]. These results showed that EGCG exhibited cardioprotective properties. The cholesterol-lowering effect of EGCG may occur through inhibition of intestinal cholesterol absorption, reduction in extracellular apoB levels, and up-regulation of LDL-C receptors by changing the mRNA expression levels of the genes involved in cholesterol metabolism in hepatocytes [100,101]. Moreover, EGCG can reduce micellar solubility of cholesterol in vitro [102].

The hypersensitivity of cardiac myofibrils to Ca^2+^ is a principal factor which leads to impaired cardiomyocyte relaxation [103]. Ca^2+^ mishandling in DCM results in depressed contractility, slow relaxation, triggered arrhythmias, and altered cellular processes, such as apoptosis or mitophagy [104]. Studies have shown that green tea catechins can be used for cardiomyopathy patients with diastolic dysfunction because of their desensitizing effects [105]. ECG and EGCG are Ca^2+^ desensitizers that directly decrease the Ca^2+^ sensitivity of cardiac myofilaments [106]. It is worth noting that hyperglycemia may give rise to diastolic dysfunction by altering the components of calcium directly, thereby impairing Ca^2+^ homeostasis [80].

Studies have shown that pretreatment with EGCG prevented the increase in mitochondrial lipid peroxidation, as well as the decrease in CAT and SOD activities and GSH content in isoproterenol-induced cardiac injury in rats [107]. Another study revealed that EGCG pretreatment protected cardiomyocytes from ischemia/reperfusion injury and increased viability of cardiac cells by reducing ROS production in H9c2 cultured rat cardiac myoblasts [108]. Adikesavan et al. reported that EGCG treatment inhibited mitochondrial lipid peroxidation and increased mitochondrial antioxidant enzymes, including CAT, SOD, GST, GPx, and GR in cigarette-smoke-induced myocardial dysfunction in rats [109]. Recent research shows that EGCG treatment suppressed arsenic-induced oxidative stress by decreasing ROS generation and MDA levels and increasing SOD and CAT activities in the mouse thymus and spleen [110]. Furthermore, EGCG treatment suppressed the increase in the levels of superoxide anion, 4-HNE, and protein carbonyl, whereas it increased the content of GSH and the activities of CAT and SOD in the diabetic myocardium [90].

Diabetic cardiomyopathy (DCM) is a serious complication of diabetes. Diabetic heart disease, in a broad sense, includes coronary atherosclerotic heart disease (coronary heart disease), diabetic cardiomyopathy, diabetic cardiac autonomic neuropathy, etc. [111]. Studies have shown that green tea catechins can be used for cardiomyopathy patients with diastolic dysfunction because of their desensitizing effects [105].

The hypersensitivity of cardiac myofibrils to calcium ions is a principal factor which leads to impaired cardiomyocyte relaxation [103]. ECG and EGCG, major polyphenols in green tea, are Ca^2+^ desensitizers that directly decrease the Ca^2+^ sensitivity of cardiac myofilaments [106]. Some studies have pointed out that EGCG has a protective effect on the hearts of patients with T2D, and it is recommended as a supplement for diabetic patients. Importantly, long-term administration of EGCG has been proven to be safe, well tolerated, and free of adverse effects [90].

##### Diabetic Nephropathy (DN)

Diabetic nephropathy is one of the most serious complications of diabetes, which can eventually lead to end stage renal disease [112]. It progresses from an increase in the glomerular filtration rate to total failure of the kidneys, passing through alterations that indicate damage to the renal glomeruli and tubules, albuminuria, mesangial expansion, fibrosis, and vascular damage [113,114]. In addition, glycogenic accumulation in the proximal tubules is a common feature in DN, and is one of the earliest signals of metabolic impairment in the organ [113,115]. Tubular alterations, with glycogen accumulation and aberrant activation of the AGE and its receptor, are considered the primary cause of proximal tubular function disturbance [115]. This, in turn, can affect glomerular function via the proximal tubule/glomerulus feedback system, and thus result in glomerular damage and the progression of DN. Therefore, any natural product that can reduce glycogen accumulation, inhibit AGE activity, and reduce oxidative damage is beneficial to improve DN.

Epidemiological research has suggested that daily consumption of green tea can reduce the incidence of diabetic nephropathy [116]. Previous researchers have speculated that the beneficial effects of green tea on proximal tubules were due to the tea’s hypoglycemiant capacity, which reduces the glycemic overload and, consequently, the AGE rate and oxidative damage [117,118].

Some experiments have shown that epicatechin may improve renal function of diabetic mice by methylglyoxal trapping to inhibit the formation of advanced glycation end-products and block inflammatory pathways. Therefore, epicatechin, as a methylglyoxal scavenger, is a potential natural product for the treatment of diabetes-related complications [119]. In a streptozotocin-induced rat model of diabetes, the intake of green tea catechins can improve renal function. The mechanism may be that green tea catechins intake can prevent glycogen accumulation in renal tubules by lowering blood glucose levels [117]. Studies have shown that oral administration of catechin-rich oil palm leaf extract (1000 mg kg^−1^) for 4 weeks can effectively alleviate renal insufficiency, glomerulosclerosis, development of fibrosis, and renal tubules in a STZ-induced diabetic rat model, all of which are associated with diabetic complications [120]. It has also been reported that EGCG, as a phytochemical with hypoglycemic and anti-fibrotic activities, can alleviate DN [121]. In a mice model with DN, the administration of EGCG reduced creatinine, glucose, and proteinuria levels [122]. Studies have also confirmed that EGCG can decrease adenine-induced serum creatinine, and improve glucotoxicity and renal injury in the animal models of DN [123].

The components of green tea can interact with proteins participating in cell signaling pathways that regulate energy metabolism, including glucose and glycogen synthesis, glucose reabsorption, hypoxia management (hypoglycemiant capacity), and cell death by apoptosis [117,118]. Such interaction reduces the accumulation of glycogen in the kidney’s proximal tubular cells in diabetes, as well as DNA damage [124]. A double-blind experiment concerning the treatment of diabetic patients with green tea polyphenols revealed that catechins could reduce podocyte apoptosis and improve kidney function by reducing microalbuminuria [125]. On the other hand, catechins can inhibit gluconeogenesis by activating the 5’ AMP-activated protein kinase [92], possibly reducing glycogenic nephrosis. In addition, EGCG can activate the protein kinase B pathway, thus enhancing cell survival and preserving nephron morphology [126]. These effects may contribute to the prevention of DN development in recent-onset diabetes [115]. In the study of the mechanism of EGCG improving DN, it was found that EGCG can significantly prevent renal oxidative damage, fibrosis, inflammation, and proteinuria caused by diabetes [121]. Raposo’s study revealed that EGCG treatment could prevent oxidative stress-induced spinal cord neurons injury in diabetic rats, indicating its potential as a nutritional preventive strategy in DN [127]. Tiwari’s research confirmed that EGCG could protect against alcoholic neuropathy in rats by inhibiting sciatic nerve oxidative stress [128]. The mechanism may be that EGCG prevents the increase in MDA content and the decrease in CAT and SOD activities and GSH content in the sciatic nerve of rats [128]. Oxidative stress-mediated Nrf2 dysfunction plays a major role in the pathogenesis of DN. Mohan’s research reveals that the beneficial effects of EGCG in mitigating DN are mediated mainly through its ability to activate the Nrf2/ARE pathway at multiple stages, i.e., by downregulating Keap1 and boosting the nuclear Nrf2 level by disrupting the Nrf2-Keap1 interaction [129]. In conclusion, catechins can contribute to the development of DN through their antioxidant properties.

## 3. Mechanisms of Catechins on Secondary Mitochondrial Diseases

In recent years, more and more scholars have recognized mitochondria as a key metabolic driver of chronic diseases such as tumor growth, and clinical trials targeting mitochondrial metabolism have achieved some success, which brings mitochondria to the forefront of tumor metabolism and immunometabolism research [130]. Since catechins can be transported to different regions of the cell, especially the mitochondria, this makes the mitochondria a viable target for the action of catechins [131].

### 3.1. Modulate Cellular Homeostasis in the Mitochondria

The intracellular redox state is a key factor regulating multiple physiological phenomena. Imbalances in intracellular redox reactions, caused by disturbances in the balance between oxidants and antioxidants, are hallmark events of multiple pathophysiological processes. Redox homeostasis is maintained by the intracellular antioxidant defense system, which is responsible for scavenging a variety of oxidants, including lipid peroxides, reactive oxygen species, and metal ions [132]. Studies have revealed that catechins can directly act as oxygen and nitrogen free radical scavengers, or exert indirect antioxidant effects by activating antioxidant enzymes and transcription factors’ activities, thereby regulating cellular redox status [133]. Due to the specificity of the mitochondrial structure, it is easily damaged by oxidation, such as protein oxidation, lipid peroxidation, and mitochondrial DNA (mtDNA) mutations [134]. Due to their antioxidant activities, including inhibition of lipid peroxidation, metal chelation, and free radical scavenging, catechins have multiple beneficial effects on chronic diseases in humans [135].

The mtDNA is susceptible to oxidative damage because of its close proximity to ROS on the inner mitochondrial membrane, its lack of protective histones, and its limited mtDNA proofreading and repair systems [136]. Overproduction of ROS can damage mtDNA from 4.4 to 48.2-fold, as much as nuclear DNA. The relatively high mutation rate of mtDNA is partly due to oxidative stress [137]. Catechins may help to maintain their own health by reducing damage to mtDNA as well as maintaining the ability of OXPHOS in mtDNA [138]. EGCG can also protect the membrane from lipid peroxidative damage and maintain the activity of membrane-bound enzymes [139].

Mitochondrial membrane dynamics, which refers to the dynamic switching between mitochondrial fission, fusion, and autophagy, are critical for maintaining mitochondrial and cellular homeostasis. The imbalance of mitochondrial membrane dynamics is manifested in increased mitochondrial fission, disappearance of membrane potential, and impaired respiratory chain function, which leads to the escape of a great number of electrons and the formation of ROS, eventually resulting in mitochondria-induced apoptosis and death [140]. The imbalance of mitochondrial membrane homeostasis can lead to the inability of damaged mitochondria to be cleaned up in time, which results in excessive production of ROS, causing excessive mitophagy and eventually leading to cell dysfunction and even death. This imbalance can also induce atherosclerosis [141]. When mitochondria are stimulated and damaged by oxidative stress, they can separate the damaged mitochondria through the process of fission, while the less damaged mitochondria can fuse with healthy mitochondria through the fusion process to continue to maintain normal functions [142]. The mitochondrial fusion process is mainly regulated by mitochondrial fusion proteins Mfn1, Mfn2, and optic atrophy protein (OPA1) [143]. Among them, Mfn1 and Mfn2 are responsible for regulating the fusion activity of the outer mitochondrial membrane, while OPA1 is responsible for regulating the fusion activity of the inner mitochondrial membrane [143]. The mitochondrial fission process is mainly regulated by the dynein-like protein (Drpl) and the fission protein 1 (Fis1) [143,144].

In a streptozotocin-induced type II diabetes rat model, EGCG administration alone or co-intervention can increase the expression levels of Mfn2 and OPA1 in the hippocampus of rats, to varying degrees. EGCG administration alone or co-intervention can also reduce the expressions of Drp1 and Fis1 to varying degrees. Although EGCG up-regulates the expression levels of Mnf2 and OPA1, EGCG does not directly induce mitochondrial biogenesis, but modulates mitochondrial biogenesis in skeletal muscle fusion. In addition, EGCG can down-regulate the expression level DRP1, which is responsible for mitochondrial fission processes [144].

### 3.2. Act Directly as Radical Scavengers

ROS are reactive oxygen species generated during mitochondrial oxidative metabolism [145], mainly including hydrogen peroxide (H_2_O_2_), superoxide anion radical (O_2_^•−^), and hydroxyl radical (^•^OH). In addition, mtDNA is close to the respiratory chain of the inner mitochondrial membrane, lacks protective histones, and has a poor ability to repair damage [146]. mtDNA is more vulnerable to O_2_^•−^ attacks than nuclear DNA, which results in mtDNA damage. Once mtDNA suffers oxidative damage, the encoding of key proteins for oxidative phosphorylation is insufficient. Naturally, it leads to production of more O_2_^•−^, which eventually leads to oxidative stress [147]. Oxidative stress is a disequilibrium status between the production and scavenging of ROS, resulting in severe damage to cellular structures, which is closely related to many metabolic diseases [148].

**Figure 1:**
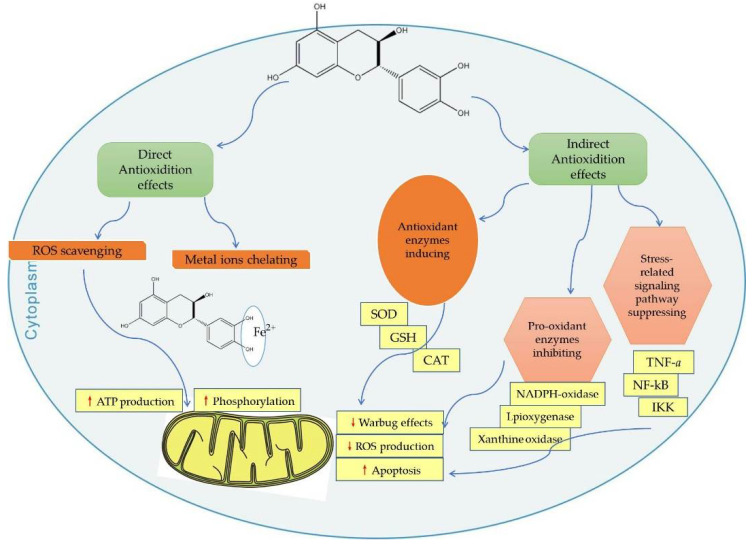
Direct and indirect effects of catechins on mitochondrial functions. CAT, catalase; SOD, superoxide dismutase; ROS, reactive oxygen species; GSH, glutathione peroxidase; TNF-α, tumor necrosis factor alpha; NF-κB, nuclear factor kappa beta; IKK, inhibitory κB Kinase. Red up arrows indicate increasing effect, and red down arrows indicate reducing effect.

Catechins act as mitochondria-targeted antioxidants that alleviate oxidative stress, which can induce deleterious cellular responses as they cross the mitochondrial phospholipid bilayer and scavenge reactive oxygen species from the source center (Figure 1) [132,149].

### 3.3. Act Directly as Metal Chelator

A large amount of evidence indicates that transition metal ions, especially iron and copper ions, act as catalysts for the oxidative damage of biomolecules and are an important factor causing oxidative stress. For example, iron overload can also directly or indirectly damage organelles, causing endoplasmic reticulum stress, and mitochondrial and autophagy dysfunction. Therefore, any compound that can chelate with related metal ions can reduce the toxicity of mitochondria [150]. Catechins are metal ion chelators, and catechins can form stable complexes with various metal ions, such as Fe^2+^, Al^3+^,Ca^2+^, Cr^3+^, Mn^2+^, and Pb^2+^.

The potential protective effect of EGCG against Cd^2+^-induced mitotic toxicity was assessed in vitro using mitochondrial-rich fractions of rat brain. It was found that EGCG completely prevented Cd^2+^-induced mitochondrial lipid peroxidation, but did not affect non-protein thiol levels. The mechanism of EGCG’s effectiveness in protecting Cd^2+^-induced mitochondrial dysfunction and lipid peroxidation may be due to its antioxidant and chelating effects [150]. It has also been experimentally confirmed that EGCG can inhibit mitochondrial membrane lipid peroxidation and cadmium-induced mitochondrial viability loss [26]. Studies have also confirmed that catechins can significantly increase cell viability by reducing intracellular ca^2+^ levels, reducing ROS formation, and improving mitochondrial membrane potential. The mechanism may be to protect calcium imbalance by regulating oxidative stress in PC12 cells exposed to lead [20].

Catechins have a strong chelating ability of divalent metal ions, due to the highest electronegativity region in the 5-hydroxy and 7-hydroxy positions of the A ring, various hydroxylations of the B ring, and the specific flavonol 3-hydroxy position of the C ring [55]. Gallocatechin, which contains more phenolic hydroxyl groups, has a stronger metal ion chelating ability than non-gallocatechin [20].

### 3.4. Protective Properties of mtDNA

Mitochondria contain a set of genetic material independent of the nucleus, namely, mitochondrial DNA (mtDNA). Studies have shown that mitochondria are core regulators of inflammation, and mtDNA may play a key role in triggering innate immune responses. When mitochondria are stressed, damaged, or dysfunctional, mitochondria expel their oxidized and cleaved mtDNA into the cytosol and subsequently into the bloodstream, triggering inflammation. Macrophages are white blood cells that reside in tissues and are responsible for detecting infection and tissue damage and recruiting other immune cells to respond. When macrophages are exposed to metabolic danger signals, the direct mitochondrial response is the rapid uptake of calcium ions from the cytoplasm, which then leads to the production of ROS, which induce oxidative mtDNA (Ox-mtDNA) production. Within the mitochondria, oxidized mtDNA is either repaired by the DNA glycosylase OGG1 or escapes through the open permeability transition pore (mPTP) in the outer mitochondrial membrane [151]. The ability of EGCG to rescue mitochondrial dysfunction and mitochondrial dynamics was evaluated by a subarachnoid hemorrhage model; the results showed that EGCG could inhibit mitochondrial dysfunction and unbalanced mitochondrial fusion under-induced by overload [Ca^2+^], as well as the neuroprotective effect in the division cycle. The therapeutic effect of EGCG suggests that it can inhibit and eliminate damaged mitochondria in time [152].

Epigallocatechin gallate (EGCG) can regulate the expression levels of a series of inflammatory cytokines. In the role of mitochondrial DNA in EGCG-mediated cardioprotection in a rat I/R model, EGCG-mediated cardiac protection can be achieved by inhibiting the release of mtDNA from damaged mitochondria [153]. In studying the role of mtDNA in the development of ventilator-induced lung injuries, EGCG significantly inhibited HTV-induced local mtDNA release and attenuated the extent of inflammatory lung injuries. In addition, the beneficial effects of EGCG on the prevention of inflammatory lung injuries were concentration-dependent [154].

Mitochondrial function has been shown to decline with age, at least in part due to oxidative damage and mtDNA mutations [155]. Studies have shown that markers of oxidative stress, such as lipid oxidation and mtDNA oxidation, also increase in the aging brain [156]. EGCG supplementation can decrease the accumulation of lipid peroxidation products in the aged brain, upregulate the antioxidant system, and enhance the activity of the electron transport chain complex in the mitochondria of the aged brain [157,158].

### 3.5. Indirect Antioxidant—Upgrade Antioxidant Enzymes

Although superoxide tends to impair cellular mitochondrial function, superoxide actually does not react with DNA and cannot cross the mitochondrial membrane. When mitochondrial DNA is damaged, in order to travel, superoxide must be converted to H_2_O_2_ either spontaneously or by superoxide dismutase. H_2_O_2_ is able to leave the mitochondria, causing damage to the entire cell. However, it is still an inefficient DNA-damaging agent, and there are multiple hydrogen peroxide scavengers located in the mitochondria to process hydrogen peroxide. Thus, intracellular oxidoreductases play an important role in maintaining normal mitochondrial function.

In fact, the lower the amount of superoxide released by the mitochondria, the higher the levels of superoxide dismutase (SOD) and glutathione peroxidase (GSH-Px), determining the maximum lifetime of the superoxide [137]. When studying the mechanism of aging, it was found that the activity of antioxidant enzymes in the body, such as SOD, CAT, and GSH-Px, decreased with increasing age, and the ability to scavenge free radicals decreased, resulting in enhanced free radical chain reactions, which also damage mitochondria function [159].

Intracellular antioxidant enzymes play a critical role in protecting cells from oxidative-stress-induced cellular damage [159]. ROS are scavenged from the mitochondria by various antioxidant enzymes (such as SOD, POD, CAT, etc.), and released into the cytoplasm, where GSH-Px or CAT perform their function [26]. Some studies have shown that catechins can activate antioxidant enzymes. The indirect antioxidant activities of catechins include activation of antioxidant enzymes, inhibition of pro-oxidative enzymes (e.g., nicotinamide adenine dinucleotide phosphate oxidase (NADPH)). They can also suppress many oxidative-stress-related pathways involved in the inflammation processes (Figure 1) [17].

## 4. Catechins and Their Regulation of the Metabolic Network Involved in Mitochondrial Function

### 4.1. Regulate Mitochondrial Function and Biogenesis

Mitochondrial biogenesis is the coordinated regulation between nuclear gene expression and transcription of mtDNA. Specifically, it is the cellular process that generates new mitochondria, as well as one of the ways in which cells adapt to changing energy requirements under various environmental and physiological conditions [160].

In fact, the process of regulating mitochondrial biogenesis is controlled by many transcription factors, such as mitochondrial transcription factor A (Tfam), peroxisome proliferator-activated receptor-*γ* coactivator 1 alpha (PGC-1α), and sirtuin-1 (Sirt-1). The master gene among them is the PGC 1-α cofactor, which coordinates the initiation of mitochondrial biogenesis from multiple stimuli. Depending on the stimulating factor, this program is carried out through a variety of signaling pathways that focus on several coactivators and nuclear transcription factors (including PGC-1α and PGC-1β) and nuclear respiratory factors 1 (NRF-1) and 2 (NRF-2) [161,162]. 

**Figure 2:**
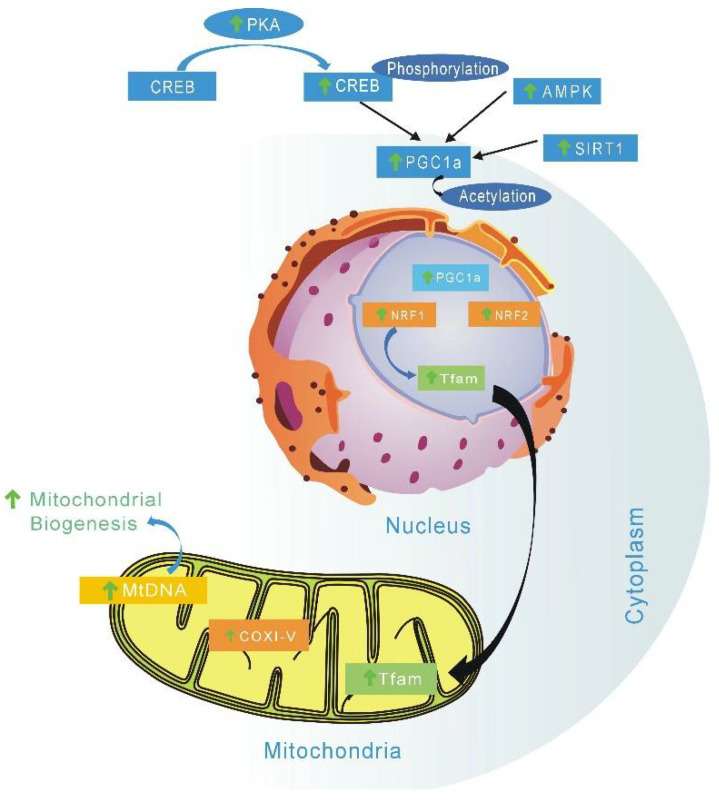
Possible metabolic mechanism stimulated by catechins to activate mitochondrial biogenesis. EGCG can increase mitochondrial biogenesis by enhancing expression of complex I, II, V, and PGC-1α. Catechins can also induce mitochondrial biogenesis by regulating critical regulators (PGC-1α, p-AMPK, SIRT1). TFAM—Mitochondrial transcription factor A; COI-V—Oxidative phosphorylation complexes I to V; mtDNA—mitochondrial DNA; NRF1/2—nuclear respiratory factors 1 and 2; PGC1α—peroxisome proliferator-activated receptor gamma coactivator 1α; SIRT1—Sirtuin 1; AMPK—AMP protein kinase; CREB—cyclic AMP response element binding protein; PKA—Protein Kinase A; ↑, vertical arrow indicates catechin induction effects.

EGCG is a mitochondrial-targeting molecule that prevents mitochondrial degradation and induces mitochondrial biogenesis by regulating critical regulators (e.g., PGC-1α, p-AMPK, SIRT1, ERRα) of mitochondrial metabolism [163]. AMP-activated-protein kinase (AMPK) is the key factor of PGC-1α involved in post-translation modification in many tissues. The phosphorylation of PGC-1α is only one of the biological mechanisms of mitochondrial biogenesis activation by AMPK [164]. AMPK is a highly conserved intracellular ATP sensor; it can be rapidly activated by low ATP levels, and then promote mitochondrial biogenesis. Studies have suggested that the main mechanism of green tea EGCG and other catechins affect energy metabolism is through the AMPK activation pathway [165]. EGCG can stimulate and improve mitochondrial function, which is due to improvement mitochondrial biogenesis and subsequent upregulation of several associated mitochondrial proteins (Figure 2) [166].

Epicatechin has been reported to activate cell surface receptors, which, in turn, stimulate endothelial cells and skeletal muscle cells, which are converted to enhance exercise capacity [167]. Relevant research confirmed that (−)-epicatechin could stimulate mitochondrial biogenesis and improve metabolism in diet-induced obesity and insulin resistance in a mouse model. Research has also found that the (+)stereoisomeric form catechins might be more efficient [168]. Sirtuins 3 (SIRT3), which is localized to the mitochondria, can regulate reactive oxygen species through different pathways; EGCG, as a targeted activating compound of SIRT3, may delay cellular senescence and inflammatory processes induced by senescence (Figure 2). SIRT3 can regulate reactive oxygen species through different pathways. While EGCG can act as a targeted activating compound of SIRT3, it may delay cellular senescence and senescence-induced inflammatory processes [169]. EGCG can also increase mitochondrial biogenesis by enhancing expression of complex I, II, V, PGC-1α, and mtDNA in 3T3-L1 adipocytes [169]. Epigallocatechin has been shown to improve mitochondrial biogenesis during a state of impaired cellular energy status, primarily by restoring the OXPHOS system and stimulating related complex (II, IV, and V) activity (Figure 2) [170,171].

### 4.2. Improve Insulin Resistance

**Figure 3:**
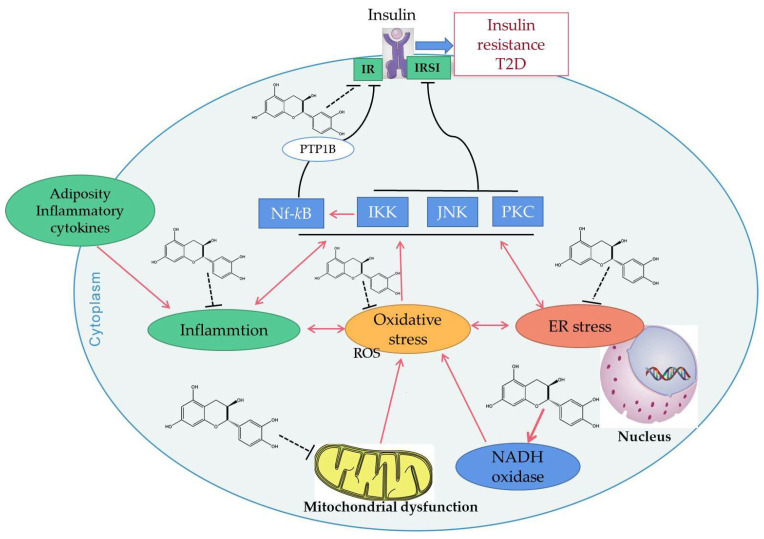
Possible mechanisms of catechins improving insulin resistance. Adiposity or inflammatory cytokines may lead to inflammation, oxidative stress and endoplasmic reticulum (ER) stress inside the cell. All of these stresses activate c-Jun N-terminal protein kinase (JNK), inhibitory κB Kinase (IKK), and the protein kinase C (PKC) signaling pathway, by which the function of insulin receptor substrates 1 (IRS1) is suppressed, decreasing its capacity to regulate downstream events in the insulin cascade. In addition, protein tyrosine phosphatase 1B (PTP1B) will be up-regulated by the activated IKK/NF-κB signaling pathway. This response will result in dephosphorylation and inactivation of the insulin receptor (IR) and insulin receptor substrates 1. Catechins have been confirmed to change the oxidative stress events by inhibiting the ROS production of mitochondria, resulting in inhibiting the activation of a series of intracellular signal pathways and reducing the occurrence of insulin resistance. Red solid arrows indicate upregulation or activation, black solid lines indicate inhibition, black broken lines indicate inhibition of catechins.

Insulin resistance (IR) refers to the decline of muscle, fat, or liver sensitivity to insulin due to various reasons, which reduces the efficiency of insulin in promoting glucose uptake and utilization, resulting in insulin resistance or insulin insensitivity. IR plays a key role in clustering risk factors for atherosclerosis, such as abnormal glucose metabolism, hypertension, and dyslipidemia [172]. Studies have shown that that IR can damage mitochondrial function and glucose metabolism, resulting in increased production of ROS [173]. When insulin production and signaling are severely impaired in the AD brain, it leads to increased oxidative stress and mitochondrial dysfunction. In turn, mitochondrial dysfunction, including overproduction of oxidants and mitochondrial loss, has been implicated in the development of IR [169]. Epidemiological data reveals that green tea consumption is inversely associated with diabetes [74]. EGCG may enhance the metabolic and vascular effects of insulin. Through experiments in obese mice, EGCG modulated hepatic mitochondrial respiratory chain complexes and demonstrated an ability to improve lipid metabolism and reduce insulin resistance in obese mice [174]. Epigallocatechin (EGC) from *Laurus nobilis* can attenuate hyperinsulinemia-induced and hyperglycemia-induced insulin resistance in HepG2 cell lines by reducing oxidative stress and improving mitochondrial biogenesis, which may have implications in drug therapy [170]. EGCG can also increase the expression of adiponectin receptor 1 (an upstream regulator of PGC-1α). For example, EGCG can improve insulin sensitivity and increase the plasma adiponectin level in spontaneously hypertensive rats [166]. Catechins have been confirmed to change the oxidative stress events by inhibiting the production of mitochondrial ROS, which results in inhibiting the activation of a series of intracellular signal pathways, thus activating the insulin receptor (IR) and insulin receptor substrates 1 (IRS1), as well as reducing the occurrence of insulin resistance (Figure 3).

### 4.3. Regulation of Calcium Homeostasis

**Figure 4:**
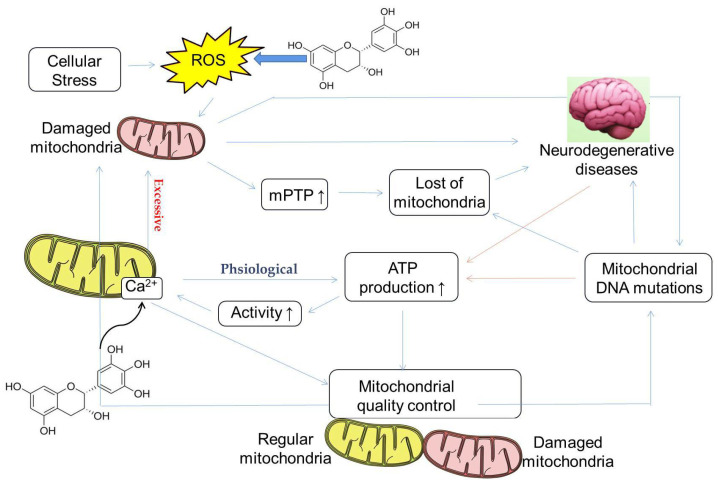
Possible mechanism of catechins regulating calcium homeostasis mitochondrial quality control involved in neurodegenerative diseases, reprinted/adapted with permission from Ref. [176]. 2020, Boyman, L. et al. As a messenger ion, Ca^2+^ plays an important role in maintaining the homeostasis of organisms and cells. The concentrations of Ca^2+^ exceeding the regular range will lead to organelle and body dysfunctions including the mitochondria, such as reducing the rate of ATP synthesis and increasing the mitochondrial membrane permeability transport pore (mPTP), decreasing mitochondrial quality, inducing mitochondrial DNA mutation, and accelerating mitochondrial apoptosis. Catechins may increase intracellular Ca^2+^ through the influx of extracellular Ca^2+^ and the release of intracellular stores, thereby maintaining mitochondrial quality control function. In addition, catechins can also indirectly reduce mitochondrial damage by reducing intracellular ROS.

Calcium ions (Ca^2+^) are involved in various physiological activities, such as blood coagulation, synthesis of neurotransmitters and hormones, and regulation of enzyme activity, and are an indispensable element of the body. The relatively stable state of Ca^2+^ concentration in mitochondria is called mitochondrial calcium homeostasis. Imbalances in mitochondrial calcium homeostasis can lead to mitochondrial dysfunction, resulting in a dramatic reduction in ATP production. At the same time, reduced mitochondrial ATP production also affects mitochondrial calcium homeostasis. When the cells are stimulated to induce Ca^2+^ influx and reach a certain concentration, mitochondria absorb Ca^2+^, and when the stimulation disappears, they release Ca^2+^, thereby buffering the violent fluctuation of intracellular Ca^2+^. Due to the fact that calcium ions play a crucial role in the physiological functions of cells, they can, for example, act as second messengers in neurons to regulate the function of synapses. Release from the mitochondria and endoplasmic reticulum increases intracellular calcium concentration, stimulating calpain, which is involved in synuclein processing and dopaminergic neuron degeneration, leading to Parkinson’s disease. The elevated calcium concentration in the endoplasmic reticulum of AD neurons causes compensatory changes in calcium signaling, which in turn affects calcium-dependent calcineurin (CaN), calmodulin-dependent protein kinase II (CaMKII), and calcium ions balance. These calcium-signaling abnormalities ultimately lead to loss of synaptic function and neuronal degeneration. Moreover, calcium influx may also lead to increased mitochondrial ROS production (Figure 4) [175].

Studies have shown that EGCG is involved in the changes of calcium homeostasis in non-excitable cells, which may be related to its effect on the Ca^2+^ transport system. In hippocampal cultured neurons, incubation with EGCG induces an increase in intracellular calcium by releasing intracellular stores (Figure 4) [177]. On the other hand, in non-excitable human U87 cells, EGCG increases intracellular Ca^2+^ through the influx of extracellular Ca^2+^ and release from intracellular stores [178]. Moreover, EGCG induces an increase in intracellular Ca^2+^ in prostate cancer cells by a multistep mechanism [179]. Recent research has found that, in human embryonic kidney cells, the effects of EGCG on Ca^2+^ homeostasis may involve the inhibition of plasma membrane Ca^2+^-ATPase, among other mechanisms [180].

### 4.4. Regulate the Epigenetic Process

Epigenetic modifications are genetic regulation models that are independent of DNA sequence, and play an important role in maintaining specific gene expression profiles. DNA methylation and histone acetylation and methylation are the main forms of epigenetic modification. Some intermediate molecules in the oxidative phosphorylation process of the mitochondrial respiratory chain (such as s-adenosyl-methionine, SAM, acetyl coenzyme A, alpha-ketoglutarate, and nicotinamide adenine dinucleotide (NAD+)), are coenzyme factors involved in the regulation of epigenetic modification enzyme activity) [181]. The mitochondrial energy metabolism disorder which accompanies aging leads to the abnormality related to the oxidative phosphorylation respiratory chain, which then affects the apparent modification state of the genome and changes the expression of related genes. For example, mitochondrial metabolic disorder affects the alpha-ketoglutarate level, which in turn regulates the activities of ten-eleven-translocation (TET) and lysine demethylases (KDM), which, finally, affects the level of DNA and histone methylation [182].

**Figure 5:**
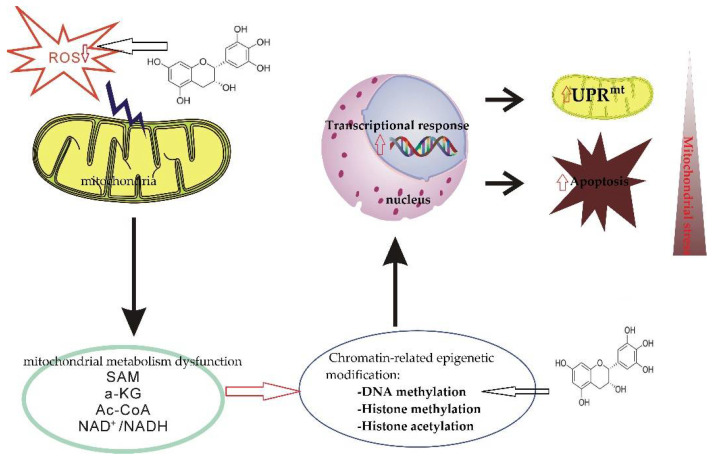
The mechanism of epigenetic abnormity and aging mediated by mitochondrial metabolism dysfunction. Mitochondrial metabolism disorders lead to abnormal levels of cellular oxidative phosphorylation and respiratory chain intermediate molecules (such as SAM, alpha-ketoglutarate, acetyl CoA, and NAD), thereby regulating the activities of related epigenetic modification enzymes, affecting the state of genome epigenetic modification, and changing related gene expression. Mitochondrial unfolded protein response (UPR^mt^) and other protective mechanisms are activated to maintain mitochondrial homeostasis. Mitochondrial metabolic disorders mediating abnormal epiregulation of gene expression is an important reason for the initiation and progression of neurodegenerative diseases. EGCG regulates DNA methylation by inhibiting activity of DNA methyltransferases.

It has been reported that catechins can modulate epigenetic processes. For example, catechins can reverse DNA methylation of tumor suppressor genes (e.g., p16^INK4a^ and Cip1/p21) and improve the transcription level of these genes [183]. EGCG regulates DNA methylation by inhibiting activity of DNA methyltransferases (DNMTs) [184]. The mechanism may be that catechins can directly or indirectly inhibit the activity of related enzymes, and decrease the expression and translation of DNMTs (Figure 5) [185]. It has also been found that each of the catechins (epicatechin, catechin, and EGCG) inhibited prokaryotic SssI DNMT- and DNMT1-mediated DNA methylation in a dose-dependent manner [186]. It has also been shown that tea polyphenols can prevent cancer by modulating DNA methylation, histone modifications, and epigenetic aberrations that occur in microRNAs [187].

### 4.5. Regulate Energy Metabolism

As mentioned above, the long-standing hyperglycemia and hyperlipidemia may induce some metabolic and molecular changes in the cells. Eventually, it will lead to metabolic complications. Therefore, any natural compounds which can alter energy metabolism will be beneficial to mitochondrial diseases. Yang’s ‘AMPK hypothesis’ proposes that AMPK activation is the main mechanism for EGCG and other catechins to influence energy metabolism [188]. The hypothesis indicates that ingestion of tea catechins suppressed gluconeogenesis and lipogenesis, and enhanced lipolysis in a coordinated manner [189,190]. It suggests that these actions of catechins are mediated by energy-sensing molecules, such as AMPK. In response to falling energy status, AMPK is activated in order to inhibit energy-consuming processes and promote catabolism to produce ATP [191,192,193]. In addition to maintaining cellular energy homeostasis, AMPK also responds to different hormone signals to maintain whole-body energy balance [193]. Tea catechins have been shown through AMPK activation to enhance the genes responsible for lipid catabolism. Targets of AMPK include enzymes such as fatty acid synthase (FAS) and acetyl CoA carboxylase (ACC) which are responsible for fatty acid synthesis [165]. Previous studies have suggested that green tea catechins can suppress adipocyte differentiation and fatty acid uptake into adipose tissue while increasing fat synthesis and oxidation by the liver, without inducing hepatic fat accumulation [194]. Overall, tea catechins can interact with proteins participating in cell signaling pathways that regulate energy metabolism, including glucose and glycogen synthesis, glucose reabsorption, hypoxia management, and cell death by apoptosis. Such interaction reduces the accumulation of glycogen in the kidney’s cells of the proximal tubules in diabetes, as well as DNA damage, and further prevents the development of metabolic complications [195].

## 5. Indirect Beneficial Effects of Catechins on Mitochondrial Disease

It is well known that normal cells can metabolize glucose into CO_2_ and H_2_O through oxygen and phosphorus under aerobic conditions, but only convert glucose into lactic acid under anoxic conditions. However, even under aerobic conditions, tumor tissue metabolizes glucose to lactate, producing only a small amount of ATP, while reducing the use of the OXPHOS mechanism, which is known as the Warburg effect. This characteristic metabolic change usually exists in cancer cells. Although the Warburg effect also produces ATP through glycolysis, many glycolytic intermediates are produced in the metabolic process. These are the precursors of anabolic processes, such as ribose-6-phosphate, NADPH, and amino acids [196].

**Figure 6:**
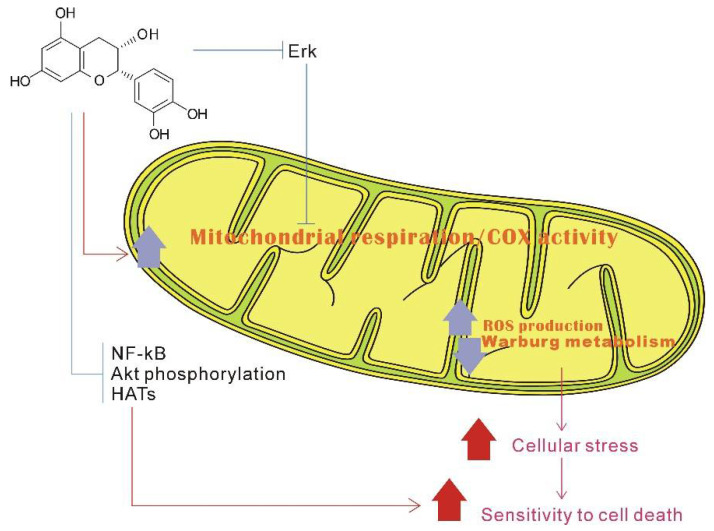
Possible model of catechin stimulates mitochondrial biogenesis and downregulates the Warburg effect, reprinted/adapted with permission from Ref. [200]. 2015, Shay, J. et al. Epicatechin may stimulate mitochondrial respiration and biogenesis, and repress the Warburg effect. At the cell signal transduction pathway level, epicatechin can also inhibit extracellular signal-regulated kinase (Erk) signaling. Through Erk and/or other signaling pathways, epicatechin can activate mitochondrial OXPHOS, which intervenes with the Warburg metabolism. Additionally, through repressing signaling pathways (such as nuclear factor kappa beta (NF-κB), protein kinase B (Akt), and histone acetyltransferases (HATs)), epicatechin can induce apoptosis in the cancer cells.

There is increasing evidence that flavonoids can regulate the activity of some enzymes responsible for aerobic glycolysis, the expression level of the translocation protein involved in glucose uptake, and the negative regulation of the Warburg effect [197]. In addition, catechins have higher inhibitory effects on lactate production and acetate dehydrogenase A as well as lactate dehydrogenase A activity. Moreover, the combination of catechin and anticancer therapeutic drugs (5-fluorouracil) showed additional cytotoxicity and induced ROS-mediated apoptosis in SNU620/5FU cells [198]. EGCG treatment can directly suppress the proliferation and migration of colorectal cancer cells. EGCG treatment of cancer-associated fibroblasts can inhibit their tumor-promoting capabilities by suppressing their glycolytic activity, which means that catechins have the ability to reverse the Warburg effect (Figure 6) [199].

Apoptosis refers to the process by which cells automatically end their lives under the control of intrinsic genetic mechanisms under certain physiological or pathological conditions. The extracellular stimuli that induce apoptosis must be transmitted through a series of intracellular signals, and the selective fragmentation of DNA between nucleosomes is one of its important hallmarks. The roles of ROS and nitric oxide in nervous system diseases, cardiovascular diseases, immune diseases, and aging are related to apoptosis to varying degrees. Recent studies have shown that changes in mitochondrial membrane function, or loss of inner membrane transmembrane potential in mitochondria, trigger various apoptosis-related metabolic changes before apoptotic cells are induced to produce DNA degradation and characteristic morphological changes [201].

Catechins perform a dual action regarding ROS homeostasis—they act as potent antioxidants under normal conditions, and as prooxidants under pathological conditions, activating the pathways which signal cell death. In other words, under normal conditions, catechins demonstrate beneficial effects protecting various cellular processes from ROS-mediated damage, whereas under pathological conditions, they can activate apoptosis and inhibit inflammation and proliferation [202]. Under pathological conditions, the EGCG can activate intrinsic mitochondrial apoptosis pathways involving the induction of the mitochondrial permeability transition pore, the impairment of mitochondrial membranes, stimulation of cytochrome c release, and activation of caspases in various tumor cells [26]. A large number of studies have confirmed the properties of catechins in inducing apoptosis of tumor and cancer cells [201,203,204,205,206].

It is well known that the mitochondria-activated apoptotic pathways are the key pathways in apoptosis [207]. The mitochondrial permeability transition pore opens in the mitochondria, thus activating the apoptosis signaling pathways [208]. Therefore, the indirect beneficial effect of catechins on mitochondrial diseases is achieved by inhibiting apoptosis of cardiomyocytes, neurons, and kidney cells. Studies have shown that EGCG suppresses H_2_O_2_-induced apoptosis through modulation of the expression of apoptosis-related genes in endothelial cells and inhibited intracellular accumulation of oxidized LDL-triggered ROS and consequent apoptosis [209,210]. EGCG can protect cardiomyocytes from ischemia/reperfusion-induced injury by decreasing oxidative stress, preventing mitochondrial damage and reducing apoptosis of cardiomyocytes [211]. In the myocardial ischemia/reperfusion model in vivo, EGCG preconditioning significantly decreased the levels of creatine kinase MB isoform and lactate dehydrogenase, increased the ATP levels, suppressed apoptosis, and partially preserved heart function [212]. Furthermore, EGCG suppressed ROS production and opening of the mitochondrial permeability transition pore, and also improved cardiomyocyte functions in the H9c2 cardiomyocyte hypoxia/reoxygenation model in vitro [212]. In many diseases, apoptosis is documented to occur through activation of the mitochondrial cyt c-stimulated caspase-3 pathway, which may be triggered by increased ROS production. Numerous studies have shown that EGCG exerts protective effects against cell injury and death. It has been reported that EGCG protects cardiomyocytes against ischemia/reperfusion-induced apoptotic cell death, both in vivo [213,214] and in vitro [215].

Furthermore, EGCG demonstrates a renoprotective effect against HFD- and STZ-induced apoptosis and fibrosis in the kidney through down-regulation of transforming growth factor beta (TGF-β), Bax, and caspase-3, and up-regulation of Bcl-2 expression in diabetic rats [216]. Moreover, some studies have further confirmed the existence of a neuroprotective effect of EGCG against β-amyloid-induced neurotoxicity in rat primary cortical neurons through increasing cell viability and Bcl-2 levels, as well as reducing the number of apoptotic cells and caspase-3 levels. This indicates that EGCG may act as a promising drug to suppress a variety of neurodegenerative disorders [217]. In conclusion, the above studies showed that catechins have an indirect effect on mitochondrial diseases due to their inhibition of apoptosis.

## 6. Discussion

In recent years, more and more research has been conducted on tea extracts for the relief and treatment of chronic diseases. Studies have shown that green tea polyphenols exhibit many disease-modifying properties, especially EGCG, which exhibits many medicinal properties, particularly neuroprotective effects [218]. However, as plant extracts are used for therapeutic drugs, their bioavailability is an issue to be considered. Bioavailability refers to the speed and extent of the drug’s absorption into the human circulatory system, which reflects the proportion of the given drug entering the human circulatory system. Therefore, before discussing the function of catechins, their bioavailability should first be assessed. The higher the bioavailability of catechins, the more effective their functions, resulting in greater health benefits [165]. Studies have shown that epicatechin is more favorably absorbed in plasma than its non-epi isomer counterpart [219]. The half-life of epicatechins is significantly longer than that of non-epi catechins, because epicatechins have a higher binding affinity to blood proteins than non-epi catechins [220]. In addition, the plasma catechin half-life is longer after oral administration than after intravenous administration due to the slower rate of absorption [220]. EGCG has higher antioxidant activity compared to other flavanol compounds [221]; the reason for this may be that EGCG is largely present in the plasma in free form (over 77%), while other catechins are associated with glucaldehyde. On the other hand, EGCG usually conjugates with sulfate and/or acid groups [222], which can be easily metabolized by gut microbiota [26].

In most analyses, ECG and EGCG showed better antioxidant activity than EC and EGC [223]. It has been suggested that since the gallic acid moiety is a key component of many flavonoids [224], the function of EGCG may be attributed to the action of the gallic acid moiety. For example, in the prevention and treatment of mitochondrial diseases, gallic acid effectively inhibits amyloid beta peptide fibril formation. It has also been suggested that the gallic acid moiety has fibril-inhibiting activity [225]. As mentioned previously, EGCG has been shown to regulate gene expression by targeting the gallic acid moiety of EGCG to DNA methyltransferases (DNMTs). Gallic acid (GA) alters methylation of lung cancer and pre-malignant oral cell lines, and significantly suppresses cytoplasmic and nuclear DNMT1 and DNMT3B within 1 week. GA is more cytotoxic to lung cancer cell lines than EGCG [185].

Gallic acid is crucial for exhibiting the glucose transport inhibition effect in gallic acid derivatives of flavonoids (catechins). The specific protein of inhibition may be glucose transporter 2. Therefore, Huijun Wang et al. propose that gallic acid derivatives of flavonoids, including ECG, EGCG, and theaflavin-3-gallate, specifically inhibit glucose transporter 2. The non-gallated flavonoids EC and EGC showed minimal 2-deoxyglucose inhibition of D-glucose transport [226]. As a phenolic compound, gallic acid has a variety of biological and pharmacological activities. More and more studies have confirmed that gallic acid provides significant protection against T2DM-mediated liver injury [227], and modulates the Nrf2 signaling pathway to protect the lungs against emphysema [228]. Gallic acid protects the cerebral cortex, hippocampus, and striatum against oxidative damage and cholinergic dysfunction [229]. Clinical researchers are convinced that the use of gallic acid with traditional anti-diabetic drugs enhances its efficiency compared with traditional drugs alone [227]. Interestingly, although the content of gallic acid in fresh tea leaves is very low, the content of gallic acid in aged Pu’erh tea is very high [230,231]. It would be of great significance to study the dynamic changes of gallic acid and its effects on chronic diseases.

In previous studies, the prodrug method has been used to improve the bioavailability of EGCG, and the experimental results have also confirmed that peracetylated-EGCG is an effective EGCG prodrug [195,232,233,234,235]. The study showed that the introduction of an alkyl group at the 4”-O position of AcEGCG led to significantly increased biological activity. Peracetylated AcEGCG could increase the amount of intracellular PGC-1α fourfold [163]. In addition to monomeric catechins, aggregated catechins also show unique health effects. For example, oolonghomobisflavan, which is isolated from oolong tea extracts, can promote health through DAF-16/FOXO transcription factors. Furthermore, oolonghomobisflavan is protective against polyQ- and Aβ-induced neuro/protein toxicity [236].

In addition to studying how to improve the bioavailability of EGCG, it is also of scientific interest to study the metabolism of catechins in the body. Some related experiments have confirmed that EGCG and its metabolites can reach the brain parenchyma through the blood-brain barrier (BBB). The above experimental results reveal that the metabolites of EGCG can play a significant role in reducing neurodegenerative diseases together with the beneficial activity of EGCG [237].

In addition to monomeric catechins, researchers found a high-molecular-weight polyphenol in black tea and oolong tea, which can significantly activate the mitochondria and increase mitochondrial membrane potential, so this polyphenol is called the mitochondrial activator (mitochondrial activation factor, MAF) [238,239]. Although the formation pathway and mechanism of MAF is unclear, it is speculated that the polymer is mainly formed in the form of catechin oxidative polymerization and catechin acidification polymerization. The recent studies indicate that MAF has functional characteristics such as scavenging free radical activity [238], improving the metabolic capacity of skeletal muscle, increasing protein synthesis in muscles [240,241], and thus increasing muscle mass [242]. Although it has been found that the DPPH free radical scavenging activity of EGCG is higher than that of MAF, the ability of MAF to activate the mitochondria is much stronger than that of EGCG, which also indicates that the mitochondrial activation ability of MAF is independent of its antioxidant activity [238]. Further research on the mechanism of MAF activation of mitochondria may be helpful for revealing the complex mechanism of mitochondrial metabolism regulation by different types of catechins.

## 7. Conclusions

Since SMD essentially refers to any abnormal mitochondrial function other than PMD, any natural product that can improve or enhance mitochondrial function may be a potential drug candidate. At present, many studies have found that catechins have protective effects on the mitochondria, and the future application prospect of catechins is also considerable (Table 1). This article reviews the characteristics of representative secondary mitochondrial diseases, as well as the inhibitory effect of catechins on secondary mitochondrial diseases and their mechanisms of action. This paper focuses on the role of catechin in regulating cell homeostasis; when catechins act as free radical scavengers and metal ion chelators, they have a protective mechanism for the mitochondria as well as a protective effect on mitochondrial DNA. We also review the indirect antioxidant mechanism of catechins, which protects the mitochondria from damage by triggering the activity of intracellular antioxidant enzymes (Figure 1). We also explain catechins and their effects on mitochondrial functional metabolic networks by regulating mitochondrial function and biogenesis, improving insulin resistance (Figure 2), regulating intracellular calcium homeostasis (Figure 4), and regulating epigenetic processes (Figure 5). Finally, the indirect beneficial effects of catechins on secondary mitochondrial diseases are also illustrated by the Warburg effect and the apoptosis effect (Figure 1). Current scientific research results indicate that catechins are an important natural compound in the treatment and prevention of mitochondrial diseases.

## Figures and Tables

**Table 1:** Possible mechanisms of catechins’ protective action in neurodegenerative diseases demonstrated in various disease models.

Experimental Objects	Drugs	Duration and Treatment Schedule	Measurement Indicators	Type of Effect	References
Transgenic drosophila melanogaster expressing Htt93Q in all neurons	EGCG	Fed with the compound of sugar supplemented with 500 mm EGCG	The number of rhabdomeres per ommatidium, motor function	EGCG reduces photoreceptor degeneration and improves the abnormal motor ability in HD transgenic flies	[243]
Male Wistar rats	EGCG	3-NP, L-arginine, andL-NAME and administered intraperitoneally to animals. EGCG was suspended in 0.05% *w*/*v* sodium carboxy-methyl-cellulose solution and administered by oral route in a constant volume of 0.5 mL/100 g of body weight	Lesion volume	EGCG treatment significantly reversed 3-NP-induced rat striatal degeneration as compared to 3-NP-treated group.	[59]
NB SH-SY5Y cells	EGCG, 6-OHDA	EGCG was added 15 min before insult with 6-OHDA for a subsequent 24 or 48 h, respectively. The inhibitor of PKC, GF 109203X, was added 30 min before treatment with EGCG.	Neuronal cell injury was evaluated by a colorimetric assay for mitochondrial function using the MTT test	Pretreatment for 15 min with EGCG conferred significant protection against 6-OHDA neurotoxicity	[62]
Hippocampal neuron from 18-day-old embryo Sprague–Dawley rats	βA (25–35) and EGCG	βA (25–100 μM) and/or EGCG (10 μM) was added to the culture medium.	Cell viability	EGCG effectively promoted the survival of βA (25–35)-treated neuronal cells.	[63]
Rat insulin-secreting RINm5F cells	EGCG from green tea	In a set of experiments, cells were pretreated with 0, 10, 20, 40 μM EGCG for 1 h prior to cytokine stimulation.	Cell viability, Insulin secretion	EGCG pretreatment prevented cells from this cytokine-induced death in RINm5F cells, with viability back to control level; EGCG can prevent the inhibitory effects of these cytokines on insulin release	[71]
Female NOD/LtJ mice	EGCG from green tea	Give either 0 or 0.05% (*w*/*v*) of EGCG in drinking-water	non-fasting blood glucose, the general clinical condition and mortality of mice	EGCG (0.05% in drinking water) significantly ameliorated hyperglycemia and delayed the onset of T1D in NOD mice	[72]
20 free-living subjects who had type 2 diabetes and took hyperglycemic drugs as prescribed	Oolong tea	Subjects consumed oolong tea (1500 mL) or water for 30 days each in a randomized crossover design. Tea was not consumed for 14 days prior to treatments.	Plasma glucose	The plasma glucose and fructosamine concentrations of diabetes patients decreased significantly (*p* < 0.001 and 0.001, respectively) after drinking oolong tea, but did not change after drinking water.	[73]
db/db mice	Dietary EGCG	Mice consumed a modified AIN-93diet containing EGCG at concentrations of 2.5, 5.0, or 10.0 g/kg of diet (EGCG 0.25%, 0.5%, or 1% w:w, n¼9/group)	Blood glucose levels	A pronounced decrease of glucose levels was observed in food-deprived db/db mice treated with EGCG	[75]
Eight-week-old obese female KK-ay and C57BL/6J mice	GTCs	Mice were treated with GTCs for 4 weeks	Plasma glucose levels	GTCs feeding decreased the blood glucose content, random blood glucose content (RBG), fasting blood glucose content (FBG), and 2-h blood glucose content (2HBG) of KK-ay mice, and increased their glucose tolerance.	[77]
Twelve pediatric cardiomyopathy patients with diastolic dysfunction	Green tea extract catechins	Oral administration for 12 months	Heart rate and blood pressure, systolic and diastolic functions, isovolumetric relaxation time, LVESD, LVEDD, LVESV	an increase in left ventricle end diastolic volume and stroke volume were observed with echocardiography	[105]
cTnT transgenic mice	Catechins	Bind EGCG to cTn subunits	Force-pCa relationships in the skinned cardiac muscle fibers	EGCG reversed the increased myofilament Ca^2+^ sensitivity of mutant mice, improved the diastolic dysfunction of the hearts of these mice, and increased their cardiac output.	[106]
Male Wistar rats	Streptozotocin (STZ), nicotinamide and (EGCG)	Oral EGCG treatment for rats induced experimental diabetes (2 mg/kg body wt)	Blood glucose, insulin, and glycosylated hemoglobin (HbA1c), serum lipid profile, the degree of cardiac apoptosis	EGCG had a positive effect against diabetes-induced cardiomyopathy by modulating the cardiometabolic risk factors, inflammation, oxidative stress, DNA damage, and cell death.	[90]
Male C57 BLKS/J genetic background (db/db) mice and their non-diabetic lean littermates (db/m; 6-week-old) and their kidneys	CE	Treated mice with CE for 16 weeks	Serum creatinine concentrations, urea levels, renal AGE levels, and morphometric changes	CE treatment for 16 weeks significantly lowered plasma creatinine and urea levels in diabetic db/db mice; CE showed notably protective effect on DN.	[119]
EA·hy926 cell line	CE	Cells were exposed to FBS-free medium containing CE (0, 250, 500, and 1000 nM) for 2 h followed by co-treatment with CE and high glucose (25 mM) for 6 or 24 h	Proinflammatory cytokines levels, IL-1β levels	CE dose-dependently abolished high glucose-induced IL-1β secretion	[119]
Male Sprague–Dawley rats	OPLE containing 1.1% (−) catechin gallate and 1.5% ferulic acid	The rats, after confirmation of diabetes induced by STZ, were treated with 1000 mg kg−1OPLE, which was dissolved in distilled water given daily for either 4 or 12 weeks by oral administration	Urinary protein concentration, Glomerular filtration rate (GFR), 8-OHdG levels in 2 h urine samples	Catechin gallate attenuated renal dysfunction (hyperfiltration, proteinuria) and suppressed increases in oxidative stress markers (8-OHdG, LPO) and the fibrotic cytokine, TGF-β1	[120]
C57BL/6 wild type mice	sodium citrate or STZ, EGCG	The diabetic mice (induced by STZ) and age-matched controls were then treated daily by subcutaneously injected EGCG (100 mg/kg) or normal saline daily, for a total period of 24 weeks.	Blood glucose, urinary albumin and urinary creatinine, renal pathological changes	The diabetic mice had a marked accumulation of fibrosis in the kidney, expansion of the mesangial matrix, and enlargement of the glomerular area, effects of which were significantly ameliorated by EGCG	[121]
Mouse podocytes	EGCG	Cells were exposed to different conditions of reagents containing varying concentrations of glucose and EGCG for 24, 48 or 72 h.	Cell viability, injury, and apoptosis.	EGCG promotes podocyte proliferation and attenuates high glucose-induced podocyte injury, reducing podocyte apoptosis induced by high glucose	[244]
The mouse hippocampal neuronal cell line HT-22	Eight tea catechin derivatives including EGCG	Cells were exposed to the indicated catechin derivative compounds for 3 h and H_2_O_2_ for 45 min	Cell viability, antioxidant properties	EGCG is the most effective polyphenol against H_2_O_2_-induced HT22 cell stress and exhibits a strong ability to reduce ROS production and radical scavenging	[223]
Wild type (WT) and inbred heterozygous β-globin knockout (BKO, muβ+/−) mice	Green tea extract (GTE) contained 24% EGCG	BKO mice were fed with a 0.2% (*w*/*w*) TMH-ferrocene supplemented diet (Fe diet) for 3 months and GTE (50 mg EGCG equivalent) for daily oral administration	Tissue iron concentration (TIC), tissue & plasma MDA (one of the lipid-peroxidation products) concentrations	GTE significantly reduced the plasma NTBI of iron-loaded BKO (*p* < 0.05) and diminished the increase of MDA	[245]
Young (3–4 months old; 15 ± 20 g) and aged (above 24 months; 420 ± 20 g) male albino rats of Wistar strain	EGCG	Rats were administered EGCG (2 mg/kg body weight/day) dissolved in saline through oral gavage for a period of 30 days	Superoxide dismutase (SOD), the activity of catalase, the level of ascorbic acid, estimation of lipid peroxidation (LPO)	EGCG supplementation resulted in the increment of the nonenzymic antioxidant status to an appreciable extent and improved the lipid peroxidation status to a considerable extent	[139]

## Data Availability

The data presented in this study are available in the paper.
